# Genome-Wide Association Studies of Milk Composition Traits in Holstein Cattle Raised in Tropical and Subtropical Conditions

**DOI:** 10.3390/ani16111652

**Published:** 2026-05-28

**Authors:** Emanueli F. P. Silva, Rita C. Gaia, Henrique A. Mulim, Luís Fernando Batista Pinto, Laiza H. S. Iung, Luiz F. Brito, Victor B. Pedrosa

**Affiliations:** 1 Department of Animal Sciences, State University of Ponta Grossa, Ponta Grossa 84010-330, PR, Brazil; emanuzootec@gmail.com (E.F.P.S.); rita@apcbrh.com.br (R.C.G.); 2 Department of Animal Sciences, Purdue University, West Lafayette, IN 47907, USA; hmulim@purdue.edu (H.A.M.); britol@purdue.edu (L.F.B.); 3 Department of Animal Sciences, Federal University of Bahia, Salvador 40026-010, BA, Brazil; luisfbp@gmail.com; 4 Neogen Corporation, Pindamonhangaba 12412-800, SP, Brazil; liung@neogen.com; 5 Biotechnology Research, Neogen Corporation, Lincoln, NE 68504, USA

**Keywords:** candidate genes, dairy genetics, fat, GWAS, production, protein

## Abstract

Genetic selection has effectively increased milk, fat, and protein yields in dairy cattle populations. This study aimed to identify genomic regions and candidate genes associated with milk composition traits in Brazilian Holstein cattle using single-step genome-wide association studies (ssGWAS), functional genomic enrichment, and gene network analyses. Phenotypic data for fat and protein yield and percentage from 2339 cows genotyped with a 100K SNP chip were analyzed. Twenty-nine candidate genes were identified, including *CPSF1*, *DGAT1*, and *SLC52A2* which were associated with all four traits. *GHR* and *NNT* were related to fat and protein percentages. Protein yield and percentage shared *HSTN*, *CSN1S1*, and *CSN2*, while *SLC15A5* and *RERG* were linked with fat yield and percentage. Functional genomic analyses indicate that these genes are mainly involved in lipid and energy metabolism and protein synthesis and secretion. Identifying such genes and pathways can support the genetic improvement of Holstein cattle under tropical and subtropical conditions.

## Simple Summary

Milk solids, including protein and fat, are important components for both human nutrition and the pricing of dairy products. Therefore, we performed genome-wide association studies to identify genomic regions associated with protein and fat yield and content in Holstein cattle raised in tropical and subtropical regions of Brazil. Phenotypic, pedigree, and genomic (100K SNP chip) data from 2339 Holstein cows raised across multiple Brazilian states were used for this study. Five of the candidate genes identified play a role in all traits evaluated, i.e., milk protein and fat production expressed in percentage and kg. In addition, 58 novel candidate genes were found to be associated with these traits. These genes are involved in key metabolic pathways such as lipid and energy metabolism, protein synthesis and secretion, hormonal signaling, cellular structure and transport, RNA regulation, and immune/stress adaptation. These findings contribute to a better understanding of the biological processes underlying variability in milk composition in Brazilian Holstein cattle.

## 1. Introduction

The milk production chain is one of Brazil’s main economic activities, with a major impact on the generation of direct and indirect jobs, and it significantly contributes to the national income [1]. In 2024, more than 35 billion liters of milk were produced by the Brazilian milk sector [2], placing Brazil in 3rd position as the world’s largest milk producer [3]. As a food, milk has a high nutritional value and provides essential nutrients for all stages of human life [4,5]. Rich in fat, protein, and other important components, its composition can vary depending on the genetics of the animals [6]. Among dairy cattle breeds, Holstein cattle (*Bos taurus taurus*) stand out for its exceptional milk yield and it is one of the most widely raised breeds worldwide [7]. Also, Holstein cattle are well known not only for their high milk yield but also for the quality of the milk they produce, particularly in terms of fat and protein content [8]. Given this production scenario, it is important to consider the factors that influence milk quality. The use of biotechnologies has made the spread of genetically superior material in different environments throughout the use of artificial insemination. With that, Holstein cows are milked across a wide range of environmental conditions and management practices, which can lead to great phenotypic variability in milk-related traits [9].

Milk fat and protein are important not only for their nutritional benefits to the human diet, but also because they directly influence the product’s pricing [10]. In this context, the market is demanding dairy products with a higher content of milk solids, which influences the production of processed dairy goods [11]. In recent years, animal breeding programs have primarily focused on increasing milk yield [12]. However, market demands have shifted toward improving milk composition, particularly the concentration of milk solids. As a result, selection strategies now aim to identify animals that not only achieve high milk production but also consistently deliver superior milk quality.

Many traits of economic importance in dairy breeding programs are polygenic, including quantitative traits such as milk yield and milk solids, i.e., multiple genes influence their phenotypic expression [13]. For a long time, animal selection was based only on phenotypic data and pedigree information. More recently, genomic data has been available on large number of animals [14], enabling more accurate genomic predictions of breeding values [15] while contributing to reduced generation intervals and greater genetic gain per time unit [12,16,17]. In this context, genome-wide association studies (GWAS) are widely used to identify genomic regions and genes controlling polygenic traits, such as milk production and quality [18,19,20]. Most GWAS involving the Holstein breed have been conducted in countries with climatic conditions and production systems distinct from those in Brazil, generally in regions characterized by mild and temperate climates, which may affect the expression of certain genes [6,21]. Therefore, the main objectives of this study were (1) to perform GWAS analyses for milk fat and protein production traits in Holstein cattle raised in tropical and subtropical regions of Brazil; (2) identify and describe candidate genes and quantitative trait loci (QTL) linked to these traits; and (3) investigate the biological pathways related to the key genomic regions associated with these traits in Brazilian Holstein cattle.

## 2. Materials and Methods

### 2.1. Phenotypic and Genotypic Data

Approval from the Animal Care and Use Committee was not required for this study as all data were obtained from preexisting databases. We used records of milk fat and protein yields and percentages obtained from 2339 Holstein cows that belonged to herds located in the Brazilian states of Minas Gerais, São Paulo, Paraná, and Rio Grande do Sul. The descriptive statistics of the data are presented in Table 1. The analyses used 305-day adjusted first-lactation records from primiparous cows milked in 2021 and 2022. All cows were genotyped using a 100K (SNP) chip panel (GGP Bovine 100K SNP Chip, Neogen Company, Lincoln, NE, USA) containing 95,526 SNPs. All 2339 phenotyped animals had genomic information available. Hair follicle samples from the animals were collected following a phenol-chloroform extraction protocol [22] were used for genotyping. A total of 3936 animals were included in the pedigree file after data editing.

### 2.2. Quality Control of the Data

The criteria used for performing the quality control of the genomic dataset included removing SNPs with a call rate lower than 0.90, SNPs located on non-autosomal chromosomes, SNPs with unknown or duplicated positions, SNPs with a minor allele frequency (MAF) lower than 0.05, and SNPs with an extreme departure from Hardy–Weinberg equilibrium (HWE; *p*-value < 10^−6^) and SNPs with high linkage disequilibrium (r^2^ > 0.995). Phenotypic records exceeding three standard deviations from the contemporary group mean were considered as outliers and removed from subsequent analyses. After quality control, 84,411 SNPs and 2339 animals remained for the GWAS [19].

### 2.3. Single-Step Genome-Wide Association Study (ssGWAS)

The ssGWAS method was employed for association analysis using the BLUPF90 family programs [23]. The RENUMF90 module was used to renumber phenotypic data, pedigree information, and genomic markers. The PREGSF90 module [24] was applied to structure the relationship matrix, followed by BLUPF90 [25] for solving the mixed model equations. Finally, the postGSF90 module was used to back-solve the genomic estimated breeding values and report the SNP solutions and approximate *p*-values for each trait. The traits were analyzed using the following animal model:

**y** = **Xb** + **Za** + **e**

where **y** is the vector of phenotypic observations; **X** is the incidence matrix relating the phenotypes to the fixed effects; **b** is the vector of fixed effects related to the contemporary group (farm, year, and season), considering animal age at first calving as linear and quadratic covariates; **Z** is the incidence matrix relating the phenotypes to the additive genetic random effects; **a** is the vector of additive genetic effects, and **e** is the vector of random residual effects. The variances of **a** and **e** were estimated using the REML approach available on the BLUPF90 package, represented as:

$$\mathrm{Var}\;\left[\begin{array}{c} a\\ e \end{array}\right]=\;\left[\begin{array}{cc} H{\sigma^{2}}_{a} & 0\\ 0 & I{\sigma^{2}}_{e} \end{array}\right]$$

where σ^2^_a_ is the direct additive genetic variance and σ^2^_e_ is the residual variance. **H** is the matrix that combines the pedigree and genomic information matrices Aguilar et al. [26]. **I** is an identity matrix. The inverse of **H** was calculated as:

$$H^{-1}\;=\;A^{-1}\;+\;\left[\begin{array}{cc} 0 & 0\\ 0 & G^{-1}-\;A_{\;22}^{-1} \end{array}\right]$$

where **A** is the pedigree-based relationship matrix; **A_22_** is the pedigree-based relationship matrix of genotyped animals, and **G** is the genomic relationship matrix based on the first method proposed by VanRaden [27]. The following algorithm was used to obtain the SNP effects [28]:

**a** = **Zu**

where **a** is a vector of breeding values for genotyped individuals; **Z** is a matrix relating individuals to the records, and **u** is a vector of SNP effects. The SNP effects were estimated using the following equation:

**û** = **IZ^′^**(**ZIZ^′^**)^−1^**â**

where **u** is a vector of SNP effects; **I** is an identity matrix; **Z** is a matrix relating individuals to the records. The *p*-values associated with the SNP effects were obtained with the POSTGSF90 program Masuda [29] based on the following equation [24]:

$$p_{i}\;=\;2\;(1\;-\;\Phi \;(\left|\frac{\alpha_{i}}{SD(\alpha_{i})}\right|))$$

where α_i_ is the estimate of the marker effects; SD is the standard deviation, and Φ is the standard normal cumulative function. SNP effects were obtained by back-solving from the (genomic) estimated breeding values of genotyped animals, and approximate *p*-values were subsequently derived from the standardized SNP effects. This approach can be used because fitting the animal as a random effect to generate estimated breeding values is equivalent to fitting all SNPs as random effects and solving these effects directly [30]. The Q-Q plots for the residuals are presented in Appendix A Figure A1.

### 2.4. Gene Annotation and Functional Enrichment Analyses

Genomic regions that contain significant SNPs (*p* < 5 × 10^−8^) were explored to identify candidate genes for the traits studied [31]. The list of candidate genes was assembled considering the exact position of the markers in the genome. The Ensembl Genes database and the ARS-UCD1.2 bovine reference genome were used for this purpose [32]. Subsequently, QTL information associated with these candidate genes was retrieved from the AnimalQTLdb (www.animalgenome.org, accessed on 19 July 2022). To improve the understanding of the biological mechanisms and processes shared by the annotated genes, functional enrichment analyses were performed using the functional annotation tools of the DAVID database [33,34]. Additionally, gene network analyses were performed using the STRING tool [35], considering known and predicted protein–protein interactions and applying a minimum interaction confidence score of 0.40.

## 3. Results

Table 1 presents the descriptive statistics for the traits evaluated.

### 3.1. Protein Yield

**Figure 1:**
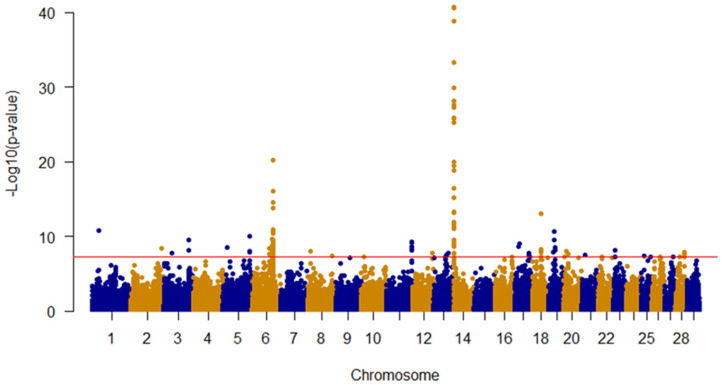
Manhattan plot of genome-wide association study (GWAS) results for milk protein yield of Holstein cows, considering an approximate *p*-value threshold of 5 × 10^−8^ (red line).

Figure 1 shows the significant SNPs for protein yield. A total of 32 significant SNPs were found on 17 chromosomes (BTA2, BTA3, BTA5, BTA6, BTA8, BTA11, BTA12, BTA13, BTA14, BTA17, BTA18, BTA19, BTA20, BTA21, BTA23, BTA25, and BTA28). There were 30 genes near these SNPs as shown in Table 2.

### 3.2. Protein Percentage

**Figure 2:**
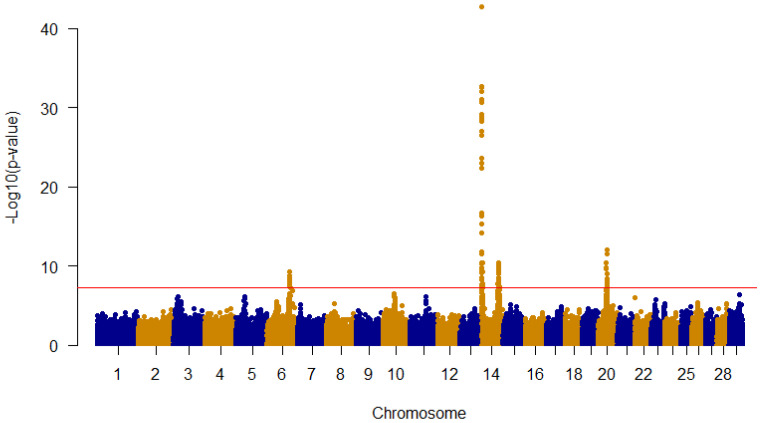
Manhattan plot of genome-wide association study (GWAS) results for milk protein percentage of Holstein cows, considering an approximate *p*-value threshold of 5 × 10^−8^ (red line).

Figure 2 shows the significant SNPs for protein percentage. The chromosomes BTA6, BTA14, and BTA20 harbored significant SNPs for protein percentage, associated with twelve main genes near these markers (Table 3).

### 3.3. Fat Yield

**Figure 3:**
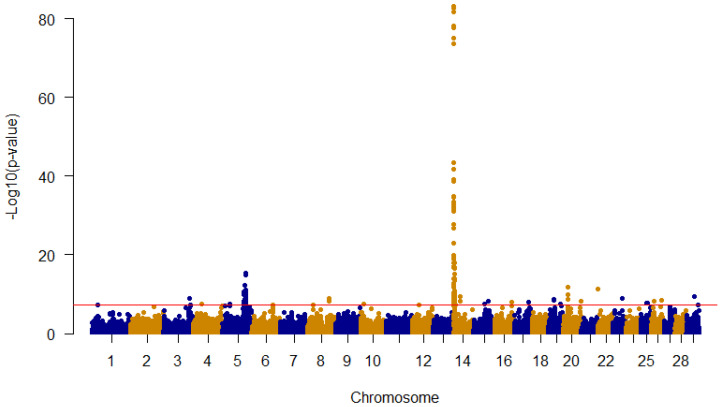
Manhattan plot of genome-wide association study (GWAS) results for milk fat yield of Holstein cows, considering an approximate *p*-value threshold of 5 × 10^−8^ (red line).

Significant markers were found on 16 chromosomes (BTA3, BTA4, BTA5, BTA8, BTA10, BTA14, BTA15, BTA16, BTA17, BTA19, BTA20, BTA22, BTA23, BTA25, BTA26, and BTA29) (Figure 3). Twenty-seven genes were found near these genomic markers (Table 4).

### 3.4. Fat Percentage

**Figure 4:**
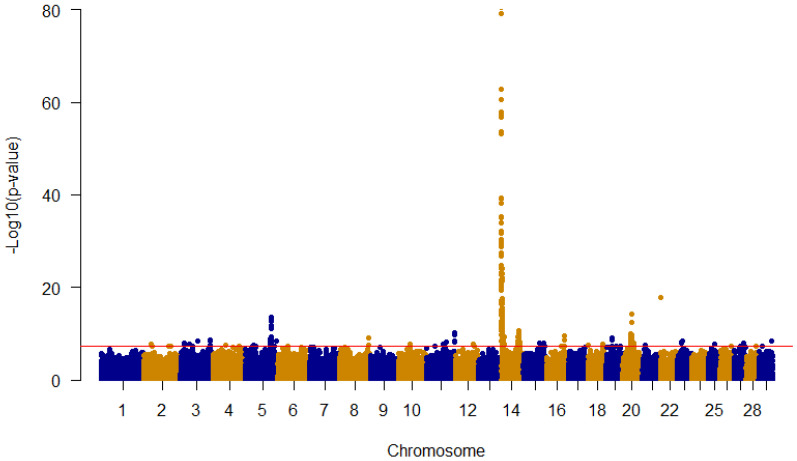
Manhattan plot of genome-wide association study (GWAS) results for milk fat percentage of Holstein cows, considering an approximate *p*-value threshold of 5 × 10^−8^ (red line).

Significant SNPs were found to be associated with variability in fat percentage. These genomic markers are located across 23 chromosomes (Figure 4), with 48 genes located in proximity to them (Table 5).

### 3.5. Functional Enrichment and Gene Interaction Network Analyses

**Figure 5:**
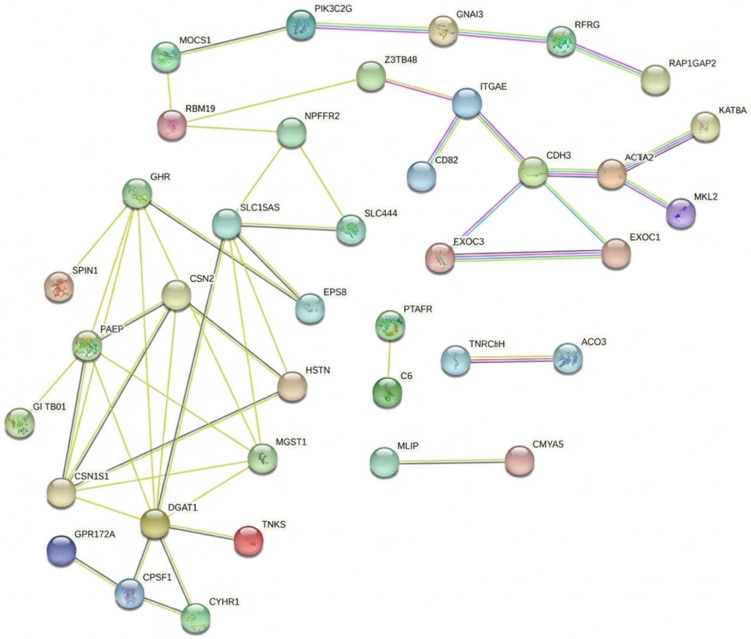
Interaction network of candidate genes for milk protein in Holstein cows.

**Figure 6:**
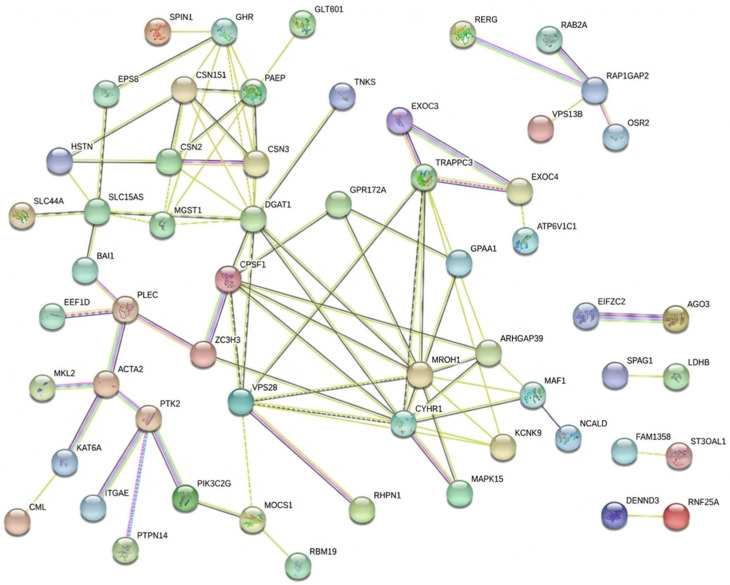
Interaction network of candidate genes for milk fat in Holstein cows.

**Figure A1:**
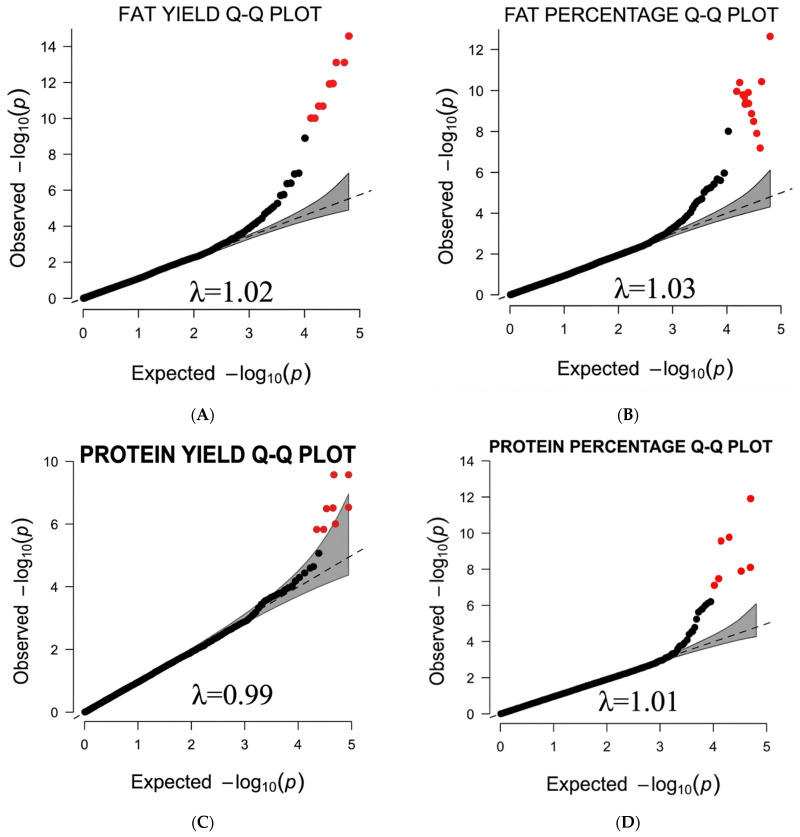
Q-Q plots for (**A**) fat percentage; (**B**) fat percentage; (**C**) protein yield; and (**D**) protein percentage.

Functional enrichment analyses enabled the identification of seven candidate genes for protein yield (Table 6 and Table A1, Appendix A), 14 genes for protein percentage (Table 7 and Table A2, Appendix A), 35 genes for fat yield (Table A3, Appendix A), and 42 genes for fat percentage (Table 8 and Table A4, Appendix A). The gene interaction networks show the relationship between the genes of interest related to the milk protein (Figure 5) and fat (Figure 6) traits.

## 4. Discussion

The identification of common genes across independent studies underscores the robustness of these findings and supports the validity of the present study. Accordingly, the use of multiple analytical approaches to identify candidate genes is essential to enhance the reliability of the results. For instance, *CPSF1*, *DGAT1*, *FOXH1*, and *SLC52A2* have been previously reported as candidate genes associated with milk quality traits [13] and were likewise identified in the present study. This concordance reinforces their biological relevance in Brazilian dairy production systems and provides additional support for the associations discussed below.

The *DGAT1* gene has been extensively studied in cattle populations from different countries, showing consistent associations with milk yield, milk composition, and fat synthesis [36,37]. The triacylglycerol synthesis enzyme DGAT1 is relevant for lipid and energy metabolism of high-producing lactating cows [38]. The significant peak detected on BTA14 (Figure 1) corresponding to the *DGAT1* locus is consistent with findings by Jayawardana et al. [39], who reported its positive association with milk protein and fat composition. Overall, *DGAT1* remains a key gene influencing milk production, protein yield, and fat percentage across cattle populations.

Similarly, the *CPSF1* gene has been associated with fat and protein percentages, as well as milk production and protein percentage, and may also affect somatic cell score [40]. Recent studies have linked the *CPSF1* gene to mRNA polyadenylation and riboflavin transport, which are essential events for protein and fat metabolism [39]. In the present study, this gene was not only identified as a candidate gene for milk fat but also influenced protein percentage and yield. These two genes were significant for all traits analyzed here and are therefore important in breeding programs aiming to improve the nutritional quality of milk, meeting market demands, and providing dairy products with greater added value.

The *GHR* gene encodes the growth hormone receptor, which is related to milk production and composition [41]. This gene has been widely studied in Holstein cattle because of its potential effect on milk production and related traits [42]. This gene is directly linked to the production of milk protein and fat, in addition to encoding the growth hormone receptor protein, which is directly related to the development and growth of tissues, including the mammary gland [43]. Furthermore, the *GHR* gene has been reported to be associated with heat tolerance in Holstein cattle [44]. This is relevant for milk production system in tropical and subtropical climate, since hotter climates tend to result in decreased milk production and altered milk composition in Holstein cows [44]. The binding of growth hormone (GH) to GHR stimulates the production of insulin-like growth factor 1 (IGF-1) in the liver. The latter is a key mediator of the growth-promoting effects of GH [45]. IGF-1 has been studied for years because of its effect on animal production. This factor is related to better productive efficiency, greater milk production, and higher milk protein and fat yield, and may play an important role in the regulation of lactation [46]. The *DGAT1* and *GHR* genes have also been reported as indicators of milk productivity and milk fat content in cows [47].

Several genes identified in this study, including *GHR*, *NRG3*, *MAML3*, and *MRTFA*, are involved in mammary gland development, cellular differentiation, and cytoskeletal organization. This pattern suggests that variation in milk yield and composition is not driven solely by metabolic pathways, but also by the structural and functional development of mammary tissue. In particular, genes related to cell fate determination, actin dynamics, and vascular development may influence the efficiency of milk synthesis and secretion. These findings support the hypothesis that genetic control of mammary gland architecture is an important component of milk quality, especially in environments where physiological challenges may compromise tissue integrity and function.

The *SLC52A2* gene was also found for all traits analyzed in the present study. An effect of this gene on fat yield, fat percentage, milk yield, protein percentage, and cheese merit has also been observed in Chinese Holstein cattle [48]. This gene is linked to riboflavin transport (GO:0032217; GO:0032218) [49,50]. Riboflavin contributes to the cellular metabolism of proteins and fats [51] and it is involved in fat metabolism in human breast and in mice. *SLC52A2* is regulated during lactation and is an important protein transporter in the lactating mammary gland [52]. The *SLC52A2* gene has been suggested to play a role in the mammary gland of dairy cows and to affect its fatty acid content [53]. Another study linked *SLC52A2* to milk production and protein percentage in Holstein cattle [54].

The *PTAFR* gene found on BTA2 is related to the immune response of cattle and their productive performance [55]. This gene was identified in a study on Holstein cattle raised in Brazil under tropical conditions and acts in bovine ovaries and granulosa cells, where it plays a role in inflammatory and immune responses that may influence reproductive and mammary functions relevant to milk production traits [56]. Furthermore, the role of *PTAFR* in uterine health has been reported, as well as its expression in the endometrial epithelium of Holstein cattle [57], suggesting its involvement in reproductive health, which can indirectly affect milk production and quality. Recent studies have linked the *PTAFR* gene to variability in bovine mastitis [58], highlighting its importance in mammary gland health and, consequently, milk quality. There is evidence that this gene is also related to the calcium signaling pathway and that its expression increases under heat stress conditions in Holstein and Jersey cattle [59]. Detection of the expression of this gene in Brazilian cattle suggests its importance for animals raised in tropical climates and indicates an essential role in the production of milk components in more extreme climates.

The recurrent detection of immune-related genes such as *PTAFR*, as well as *NPFFR2*, *TRAPPC9*, and *CCL28* indicates that immune competence is closely intertwined with milk production and quality in this population. Beyond their classical roles in inflammatory response and pathogen defense, these genes may indirectly influence milk yield and composition by modulating mammary gland health and resistance to mastitis. In tropical environments, where pathogen pressure and thermal stress are elevated, the genetic ability to maintain immune balance is particularly relevant. This reinforces the concept that selection for milk quality should also consider immune resilience as an integral component of productive efficiency.

The *MRTFA* gene was found as a candidate for protein yield in Holstein cattle. This gene interacts with the Myocardin transcription factor (responsible for the production of contractile proteins), contributes to the differentiation of smooth muscle cells [60], and might be linked to actin, which is present in mammary tissue cells and responsible for moving milk through the mammary ducts and canals during milking. As observed in the present study, the *MRTFA* gene has also been reported as a candidate gene for milk and protein production in multiple cattle breeds, including Holstein, Angus, Brahman, and crossbred animals, with samples collected from various geographical regions in North America and Europe [61]. The *CSN1S1* and *CSN2* genes belong to the casein family, located on bovine BTA6. Caseins represent the major milk proteins and are encoded by four distinct genes—*CSN1S1*, *CSN1S2*, *CSN2*, and *CSN3*—which play key roles in the nutritional value and functional properties of milk [48].

The *CSN1S1* gene showed notable relevance in this study. It is known to be directly regulated by progesterone and estradiol, and exhibits antioxidant activity as well as protein-stabilizing functions [50,51]. Moreover, *CSN1S1* has been associated with cytoplasmic activity and the membrane of the lactating mammary gland, and it may influence the female reproductive system and the phosphate and calcium transport capacity of milk [52]. Previous studies have linked *CSN1S1* to protein and fat production, and milk yield [53,54]. The CSN2 gene encodes β-casein, a major milk casein protein comprising 30–35% of total caseins, which influences casein fraction composition and thereby optimizes dairy product yield, cheese-making properties, and milk processing efficiency [56].

The *CSN3* gene, encoding κ-casein, is of particular importance for the dairy industry, as it plays a central role in determining milk production and compositional properties [48,58]. κ-casein stabilizes casein micelles, aggregates that are essential for the structural and functional integrity of milk proteins [59]. Unlike other caseins, κ-casein directly determines the ability of milk to form a proper curd, making it a key factor in cheese production and, consequently, industry profitability through improved protein synthesis efficiency, milk price, and product yield [57]. Interestingly, while studies have shown that mild heat stress can reduce overall cow performance, the expression of *CSN3* and *CSN2*, and thus κ-casein and β-casein, was not significantly affected by heat stress in Holstein cattle raised in Brazil [60]. This suggests that κ-casein production may be more resilient to environmental challenges, highlighting its unique importance for milk quality under tropical conditions.

The identification of multiple casein genes (*CSN1S1*, *CSN2*, and *CSN3*), together with *HSTN*, reinforces the central role of casein synthesis in determining milk quality in Brazilian Holstein herds. Interestingly, previous studies have reported that the expression of κ-casein and β-casein is relatively stable under heat stress conditions, suggesting a certain degree of resilience of these proteins to environmental challenges. This may represent an adaptive advantage in tropical production systems, where maintaining milk solids content under thermal stress is economically critical. The present findings support the relevance of these genes not only for technological properties of milk, but also for production stability in challenging environments.

The *HSTN* gene was previously reported as a candidate gene for both protein percentage and protein yield in the present study. This gene is related to the expression of beta-casein and, like the other casein genes, is regulated during lactation [62]. Furthermore, although there are only a few studies on the *HSTN* gene, it was also reported as a candidate for milk quality [62].

The *NPFFR2* gene encodes a receptor for neuropeptides, influencing the energy balance, and was found to be a candidate for protein yield [19]. In addition, the protein can be found in the plasma membrane and in actin filaments and has been linked to immune responses and mammary gland cells [49,50,63]. Actin filaments are important for cell contraction and contribute to the secretion of milk in the mammary gland [64]. Since *NPFFR2* is related to immunity, this gene is also important for resistance to mastitis [65]. Furthermore, the *NPFFR2* gene has been linked to cow longevity, milk production, and milk components in Ayrshire, Brown Swiss, Guernsey, Holstein, and Jersey [66]. It was also considered a candidate gene for polyunsaturated fatty acids [38]. The latter are essential nutrients that play a vital role in human health. For this reason, this gene is relevant in the present study since studies have demonstrated its importance for milk quality and efficient animal production.

The *SCARA5* gene plays a key role in the cellular response to heat stress (GO:0034605), being linked to changes in the state or activity of a cell in response to a temperature stimulus [49,50]. Recent studies have also associated this gene with the biological process of the response to heat stress in cattle, including both indigenous Ethiopian breeds and European beef breeds. In these studies, heat stress was assessed using environmental temperature and humidity measurements, and physiological indicators such as increased rectal temperature and respiration rate, confirming that the animals were indeed exposed to heat stress conditions [67]. It is important to link genes to heat effects since dairy cows exposed to heat stress tend to produce less milk because of reduced feed intake, increased water and electrolyte losses, and negative effects on mammary physiology [68]. The *SCARA5* gene is regulated by female hormones and maternal signals [69] and is expressed in stromal tissue during pregnancy; after parturition, its expression shifts predominantly to immune cells [70]. This expression pattern supports a potential role of *SCARA5* in physiological processes related to lactation and milk production. Since this gene is related to heat response in cattle, it is important to correlate its effects in dairy cattle raised in tropical and subtropical climates in Brazil.

An important and distinctive aspect of the present study is the consistent identification of genes associated with heat stress response and thermal adaptation, such as *SCARA5*, in addition to other genes such as *NNT* and *VPS28*. Unlike most GWAS conducted in temperate environments, the animals evaluated here were raised under tropical and subtropical conditions, where heat stress is a chronic and biologically relevant challenge. The detection of these genes suggests that, in this population, part of the genetic architecture of milk production and composition is shaped by environmental pressure. This reinforces the concept that selection for milk quality in tropical systems cannot be fully extrapolated from studies conducted in cooler climates, as adaptive mechanisms related to cellular stress response, immune function, and metabolic stability appear to play a central role in sustaining lactation under thermal challenge.

The *MAML3* gene is related to Notch receptors (GO:0007221) in mammals. These receptors contribute to cell–cell communication, i.e., it is necessary for signaling of the fate of cells such as vasculogenesis and hematopoiesis [49,50,71,72,73]. For this reason, this gene may have an indirect effect on lactation since good blood vessels and blood containing nutrients that passes through the mammary gland cells are necessary for milk production [74].

The *TRPV3* gene found on BTA19 is related to calcium channel activity (GO:0005262) with focus on major dairy breeds like Holstein, but the Gene Ontology (GO) annotation applies broadly across the entire *Bos taurus* species, regardless of breed [49,50]. Furthermore, *TRPV3* has been reported to contribute to ion transport, particularly in tissues involved in calcium absorption, such as the intestine and kidney in cattle. In these tissues, TRPV3 channels play a role in regulating calcium uptake, which is essential for maintaining calcium homeostasis and preventing metabolic disorders like milk fever in cattle [75]. A rapid decline in blood calcium (Ca^2+^) levels is commonly observed during calving. This phenomenon appears to be associated with the difficulty in mobilizing a sufficient amount of Ca^2+^ from bones [76]. Decreased levels of Ca^2+^ can cause hypocalcemia and increase the risk of other metabolic diseases, such as ketosis and abomasal displacement.

The *SLC30A5* gene is related to zinc transport and the sequestration of zinc vesicles that are secreted in the mammary gland. Its action is also linked to hormones related to milk secretion in mice [77]. Kumar et al. [77] also reported that reduced *SLC30A5* levels lead to lower zinc levels in breast milk. This gene has also been shown to influence the transport and absorption of dietary zinc during the embryonic phase [78]. Zinc is essential for various physiological functions, including the development and functioning of the immune system, growth, and reproduction, as well as for protein synthesis [79]. We therefore suggest that the *SLC30A5* gene exerts an indirect effect on bovine milk production since the immune system helps prevent diseases in dairy cattle that can affect productivity and quality.

The *NRG3* gene is implicated in the early stages of mammary gland organogenesis, contributing to mammary system formation and subsequent mammary epithelial structure development in cattle (GO:0060596) [49,50]. In humans, this gene has been reported to influence mammary gland development [80]. *NRG3* has also been described as important for the development of the mammary gland in cattle [81]. This association may also be linked to the production of milk fat and protein, which contribute to milk quality since good levels of milk solids require adequate development of the mammary gland.

The nicotinamide nucleotide transhydrogenase (*NNT*) gene, which was found as a candidate gene for fat and protein percentages in the present study, is associated with a protein of the inner mitochondrial membrane. This gene is involved in cellular energy metabolism. The gene has been reported to have an important effect on the regulation of mitochondrial NADPH levels, mitochondrial functions, oxidative stress, and of ATP production [82], highlighting its importance in metabolic processes relevant to milk composition. Although there are few reports on NNT expression in cattle, studies in sheep have linked this gene to immune response and heat stress adaptation. Given that the animals from the present study were exposed to heat stress, the potential role of NNT in stress response is relevant to our findings [83]. The *SLC4A4* gene was found to be important for protein percentage in the present study. It functions as a glucose transporter, and glucose uptake by mammary gland is essential for milk production [84]. The *SLC4A4* gene has been described as important for both milk and protein production, further supporting its relevance in dairy cattle [85].

The *PLEC* gene was found to be significant for protein percentage and yield. The gene is located close to *DGAT1*, which affects milk volume and fat percentage in cattle. Furthermore, the biological function of the *PLEC* gene is associated with the maintenance and stability of cells, cell migration, and the formation of physical barriers [86]. There is further evidence of a strong relationship of this gene with protein production in Holstein cattle [84]. The *PLEC* gene, together with the *VPS28* gene, have been described to influence milk production and protein and fat percentages, in Chinese Holstein [87]. The *PLEC* gene was also found as a candidate for milk production alongside *DGAT1* and *TRAPPC9*. It is noteworthy that the *PLEC* and *TRAPPC9* genes were also detected in Chinese Holstein cattle raised under tropical and subtropical conditions [88].

In addition, expression of the *EPPK1* gene, which encodes epoplankin, has been reported in response to heat stress and is associated with changes in unsaturated fatty acids in dairy cattle [38]. The *VPS28* gene has been shown to be expressed in the mammary gland of Chinese Holstein cattle and is associated with milk fat production [89]. This gene has also been linked to milk production under heat stress conditions, with the protein being involved in heat shock resistance [90]. Furthermore, the *VPS28* gene plays a role in cellular processes within the mammary gland and in milk production [91]. The adhesion G protein-coupled receptor B1 (*AGRB1*) gene has transmembrane signaling receptor activity and has been linked to lactose production in cattle [92]. This gene was considered a candidate gene for urea nitrogen in milk. A decrease in urea nitrogen enables improvement of the health of cows and reduces the environmental footprints of the dairy sector. In addition, this gene has been related to protein metabolism or binding and angiogenesis (blood vessel formation) [93]. Understanding the action of genes in dairy cattle is fundamental for genetic improvement and for enhancing desirable traits. The use of genomic tools permits the identification of cattle with more favorable alleles, contributing to the development of more productive, healthy herds adapted to the needs of the dairy sector. The genes described can have a direct or indirect effect on milk production since all of them contribute in some way to lactation.

Related to zinc, the *ZC3H3* gene is known to play different roles in cellular processes and has been reported as candidate for milk fat and protein percentages and milk production [39,94]. The *TRAPPC9* gene is relevant for the regulation of cell differentiation and control of immune system mechanisms [38,95] and has therefore been indicated as a candidate gene for resistance to mastitis [95], consequently contributing to milk quality. The gene has also been reported as a candidate for milk production traits in Holstein cattle, showing higher expression in the mammary gland of lactating cows compared to other tissues [96]. Recent studies corroborate the information described above by showing that the *TRAPPC9* gene is related to bovine mastitis, is more expressed in the mammary gland, and is linked to the immune system [97].

Studies have linked the *CPSF1* gene to variability in fat yield and percentage in Holstein cattle, with a significant effect on both milk and fat production [40]. In a study by Bakhshalizadeh et al. [98], this gene was reported for milk fat percentage [98]. Supporting evidence from another study published the same year further confirmed its role in regulating fat synthesis and deposition [99]. The palmitoyl-protein thioesterase 1 (*PNPT1*) gene plays a structural role in bovine ovarian follicles, contributing to the organization and development of mature oocytes [100]. In addition, this gene has been associated with thermal adaptation in cattle [101] and was related to fat yield in the present study. In cattle, the *ENSBTAG00000022427* gene was identified near *TENM4* and plays a role in the embryonic mesoderm [102]. It is important to relate the effects of the genes identified in the present study to findings from previous studies to demonstrate their relevance to bovine physiology and their potential contribution to improving lactation quality. For example, genes associated with reproductive processes may influence gestational and prepartum hormonal dynamics that are essential for mammary gland development and subsequent milk production [88]. Furthermore, identifying genes associated with thermal adaptation is particularly relevant, as Holstein cattle raised under tropical and subtropical conditions in Brazil may harbor genetic variants that influence milk production under these environmental constraints.

The *LRP2* gene has been extensively studied in dairy cattle. *LRP2* encodes low-density lipoprotein receptor-related protein 2 and has been associated with metabolic diseases [103] and lipid metabolism. This is particularly interesting since energy imbalances can affect lipid metabolism, a fact that renders this gene a candidate for investigating the metabolic effects of negative energy balance [104]. Metabolic diseases and a negative energy balance can result in a decline in milk production, compromising the profitability and efficiency of dairy production. Therefore, this gene influences lactation and consequently milk composition. The physiological regulation of the *LRP2* gene expression in the mammary tissue of dairy cows provides further support for the specific role of this gene in mammary gland development and function [105]. Higher expression of the *LRP2* gene is observed in human and bovine colostrum compared to milk, with the gene playing important roles in metabolism, embryonic development, and control of the immune response [106]. In the current study, the *LRP2* gene was found as a candidate gene for fat percentage.

Studies have shown expression of the *EXOC4* gene in Angus and Charolais cattle, as well as in the Holstein breed, which was associated with gestation length [107]. The *NELL2* gene was related to fertilization and embryonic development in dairy cows exposed to heat stress [108]. *NELL2*, which is known to promote the formation of new blood vessels (angiogenesis), was down-regulated in response to the increase in milking frequency [109].

The *FAM13A* gene exerts a constant effect during first and second lactations but has more pronounced effects during mid-third lactation [110]. Therefore, it may play a key role in the regulation of milk production throughout lactation. The *FAM13A* gene has also been shown to be involved in susceptibility to mastitis in Jersey cows [111]. One study investigated the relationship between urea concentration and expression of the *BRINP1* gene in bovine endometrial cells, indicating it as a candidate gene [112]. A balanced plasma urea concentration has several benefits for dairy cows, being an indicator of good metabolic health, favorable milk production, and good fertility rates. On the other hand, abnormal urea levels may be associated with health problems such as ruminal acidosis, nutritional imbalances, and reduced reproductive efficiency. Cows with health problems produce less milk with lower fat and protein levels. The *CD82* gene has been associated with immature and mature oocytes in cattle and was expressed in ovarian tissue [113]. Embryonic development and lactation are interconnected, with adequate development of the embryo being essential for the normal occurrence of lactation. Furthermore, this gene was upregulated in heifers with low fertility [114]. The *CD82* gene has also been linked to gene expression in the mammary gland [115], an organ that is essential for lactation, playing vital roles in milk production, secretion, and composition. The molybdenum cofactor synthesis 1 (*MOCS1*) gene [116] has been associated with fat and protein percentages in Holstein cattle [19].

The *CCL28* gene has been linked to lactation persistence in Holstein cattle and is essential for the guiding and accumulation of antibody-secreting cells in the mammary gland during the lactation period [117,118]. This gene has also been associated with milk protein percentage and mastitis control; thus, it may indirectly affect milk production [84]. A link of this gene to the immune response has also been reported [119]. The *C6* gene has been associated with immune response-related traits and with cattle fertility. Furthermore, this gene was found to be involved in antibody production and in the phagocytosis of bacterial cells in the mammary gland [120]. The cow’s immunity plays a crucial role in lactation, affecting the animal’s health, milk quality, resistance to diseases, well-being, and longevity. Thus, these genes indirectly influence lactation and milk composition.

The genes described are involved in the synthesis and regulation of milk components and are important for cellular balance and adequate body function. Understanding the effect of genes and their expression is crucial for the use of this information in genetic improvement programs to increase the productivity of Holstein cattle herds in Brazil.

Our results indicated potential novel candidate genes for Holstein cows reared under tropical and subtropical conditions, paving the way for future functional studies. These genes include *CDH3*, *GNAI3*, *LRRC1*, *MLIP*, *MRTFB*, *RGMA*, *TNRC6B*, *ZBTB46*, and *ZNF691*, which are related to protein production, as well as C25H16ORF82, OPCML, OR2D37, RERG, and RNF1144B, which are related to fat production. Although functional information for some of these genes is still limited, their detection in animals raised under tropical and subtropical conditions suggests that they may be involved in adaptive pathways specific to these environments. These findings open new perspectives for functional genomic studies and reinforce the importance of investigating non-temperate populations to uncover novel components of the genetic architecture of milk production.

In summary, the candidate genes identified in this study can be classified into levels based on the strength of supporting evidence. Some reported genes have strong evidence and represent a robust association with milk fat and protein, such as. *DGAT1*, *CPSF1*, *SLC52A2*, *GHR*, *CSN1S1*, *CSN2*, *CSN3*, *LRP2*, *TRAPPC9*. This study also presents some exploratory findings that require further validation, including *CDH3*, *GNAI3*, *LRRC1*, *MLIP*, *MRTFB*, *RGMA*, *TNRC6B*, *ZBTB46*, and *ZNF691*. This distinction strengthens the interpretation of the present results by separating well-established signals from novel candidates potentially related to adaptation under tropical production systems.

## 5. Conclusions

GWAS analyses enabled the identification of genomic regions associated with variability in milk fat and protein traits in Holstein cattle raised in Brazil. The *CPSF1*, *DGAT1*, and *SLC52A2* genes were identified as candidate for all four traits analyzed. Furthermore, the *SLC52A2* and *DGAT1* genes exhibited the most prominent peaks in the Manhattan plot. Regarding functional genomic analyses, the *CSN1S1*, *CSN2*, *PLEC*, *MAML3*, *MRTFA*, and *MRTFB* genes were found to be involved in biological processes related to protein yield. For protein percentage, the *EPPK1*, *PLEC*, *VPS28*, *ADGRB1*, *CSN1S1*, *CSN2*, *CSN3*, *MAPK15*, *TRAPPC9*, *GHR*, *HSTN*, *CPSF1*, *ZC3H3*, and *SLC4A4* genes also exhibited functions related to biological processes that might impact milk quality traits. These findings highlight the importance of these genes for the determination of protein yield and protein percentage in bovine milk. Regarding fat yield, the *PLEC* gene was highlighted, followed by *PTK2*. On the other hand, regarding fat percentage, *AGO2* and *AGO3* genes are involved in mRNA cleavage, as well as functions related to RNA and biological and cellular processes. These findings highlight the importance of these genes for the regulation of fat production and composition in bovine milk.

Some genes associated with milk production traits were found exclusively in Brazilian Holstein herds, including *AGO2*, *AGO3*, *C25H16ORF82*, *CDH3*, *EEF1D*, *ENSBTAG00000025756*, *ENSBTAG00000038214*, *ENSBTAG00000048091*, *ENSBTAG00000051599*, *ENSBTAG00000053188*, *ENSBTAG00000054671*, *ENSBTAG00000055067*, *GNAI3*, *GPAA1*, *GRHL2*, *IVNS1APB*, *KCNA4*, *LRRC1*, *MAPK15*, *MLIP*, *MRTFB*, *NADSYN*, *OPCML*, *OR2D37*, *OSCP1*, *PTK2*, *RAP1GAP2*, *RERG*, *RGMA*, *RNF1144B*, *RTRAF*, *SH2D4B*, *SPAG1*, *STK40*, *TNRC6B*, *UBXN2A*, *ZBTB46*, and *ZNF691*. The present findings highlight the importance of genes in regulating fat production and composition in bovine milk. Understanding genetic processes and identifying genes of interest expressed under specific environmental conditions enable more efficient genetic improvement of animals by targeting adaptive traits and optimizing productive performance in challenging environments.

## Figures and Tables

**Table 1:** Phenotypic means and standard deviations (SD) for protein yield (PY_kg), protein percentage (Prot_%), fat yield (FY_kg), and fat percentage (Fat_%), along with estimates of additive genetic variance (σ^2^_a_), phenotypic variance (σ^2^_p_), heritability (h^2^), and the standard errors of heritability estimates.

Trait	Mean (SD)	σ^2^_a_	σ^2^_p_	h^2^ (SE)
PY_kg	275.8 (52.75)	634.51	2791.02	0.23 (0.02)
Prot_%	3.04 (0.27)	0.044	0.078	0.56 (0.03)
FY_kg	307.43 (59.67)	917.44	3636.58	0.25 (0.02)
Fat_%	3.3 (0.31)	0.102	0.188	0.54 (0.03)

**Table 2:** Description of the main SNPs for milk protein yield in Holstein cows.

CHR	Position (bp)	−log_10_ (*p*-Value)	Genes	Number of QTL
BTA2	125,215,960	8.43	*PTAFR*	-
BTA3	103,393,004	8.12	*ZNF691*	-
BTA3	33,860,700	7.80	*GNAI3*	-
BTA5	111,807,008	10.03	*MRTFA*	2
BTA5	111,553,050	8.02	*TNRC6B*	1
BTA6	85,424,759	20.19	*CSN1S1*	22
BTA6	85,450,410	16.06	*CSN2*	55
BTA6	87,314,427	9.03	*NPFFR2*	9
BTA6	85,470,165	8.83	*HSTN*	2
BTA8	10,929,511	8.01	*SCARA5*	-
BTA8	100,103,184	7.33	*SVEP1*	-
BTA11	103,257,948	9.14	*PAEP*	70
BTA12	80,653,811	7.71		-
BTA13	54,027,332	7.49	*ZBTB46*	2
BTA14	609,870	40.72	*DGAT1*	304
BTA14	550,784	40.59	*CPSF1*	12
BTA14	580,019	40.56	*SLC52A2*	1
BTA14	487,527	27.65	*CYHR1*	20
BTA14	2,019,390	25.94	*TSNARE1*	35
BTA17	18,980,582	9.07	*ENSBTAG00000055067*	-
BTA17	17,698,911	8.68	*MAML3*	13
BTA17	60,492,777	7.73	*RBM19*	1
BTA17	60,795,229	7.44	*RBM19*	1
BTA18	35,979,599	7.92	*CDH3*	-
BTA19	24,262,976	10.59	*TRPV3*	-
BTA20	10,663,562	8.02	*SLC30A5*	-
BTA20	16,639,450	7.62	*ENSBTAG00000053188*	-
BTA21	13,990,258	7.47	*RGMA*	-
BTA23	6,493,652	8.08	*MLIP*	-
BTA23	6,756,584	7.30	*LRRC1*	-
BTA25	13,272,209	7.41	*MRTFB*	-
BTA28	37,297,081	7.66	*NRG3*	42

**Table 3:** Description of the main SNPs for milk protein percentage of Holstein cows.

CHR	Position (bp)	log −10 (*p*-Value)	Genes	Number of QTL
BTA6	85,470,165	8.74	*HSTN*	2
BTA6	85,451,221	8.63	*CSN2*	55
BTA6	85,274,240	8.35	*ENSBTAG00000038214*	-
BTA6	85,424,759	8.14	*CSN1S1*	22
BTA6	86,618,893	8.10	*SLC4A4*	16
BTA14	580,019	42.72	*SLC52A2*	1
BTA14	609,870	32.63	*DGAT1*	304
BTA14	550,784	32.59	*CPSF1*	12
BTA14	2,019,390	30.65	*TSNARE1*	35
BTA14	487,527	28.63	*CYHR1*	20
BTA20	32,018,037	11.99	*GHR*	168
BTA20	31,178,183	10.38	*NNT*	12

**Table 4:** Description of the main SNPs associated with variability in milk fat yield in Holstein cows.

CHR	Position (bp)	−log_10_ (*p*-Value)	Genes	Number of QTL
BTA3	106,010,224	8.93	*PPT1*	-
BTA4	36,408,002	7.55	*SEMA3A*	-
BTA5	93,516,066	15.45	*MGST1*	11
BTA5	93,627,442	15.35	*SLC15A5*	5
BTA5	88,557,863	8.17	*LDHB*	-
BTA5	94,720,983	7.81	*RERG*	6
BTA5	94,308,747	7.35	*EPS8*	16
BTA8	89,675,148	8.24	*SPIN1*	1
BTA8	90,078,400	8.15	*ENSBTAG00000025756*	-
BTA10	10,746,309	7.45	*CMYA5*	1
BTA14	580,019	83.04	*SLC52A2*	1
BTA14	550,784	83.03	*CPSF1*	12
BTA14	609,870	83.03	*DGAT1*	304
BTA14	2,019,390	82.72	*TSNARE1*	35
BTA14	487,527	77.71	*CYHR1*	20
BTA15	46,100,916	7.52	*OR2D37*	-
BTA16	69,148,336	8.09	*PTPN14*	-
BTA17	60,492,777	8.02	*RBM19*	1
BTA19	25,250,953	8.43	*KIAA0753*	-
BTA20	16,257,634	11.71	-	-
BTA20	16,311,925	9.78	*ENSBTAG00000053188*	-
BTA20	16,639,450	8.76	*ENSBTAG00000053188*	-
BTA20	71,674,625	8.16	*EXOC3*	-
BTA22	52,575	11.37	*ENSBTAG00000053760*	-
BTA23	39,111,036	9.03	*RNF1144B*	-
BTA25	24,615,711	7.86	*C25H16arf82*	-
BTA25	27,555,271	7.72	*AHSP*	-
BTA26	10,653,831	8.22	*ACTA2*	-
BTA29	34,333,891	9.35	*OPCML*	-

**Table 5:** Description of the main SNPs for milk fat percentage of Holstein cows.

CHR	Position (bp)	−log_10_ (*p*-Value)	Genes	Number of QTL
BTA2	27,018,016	7.84	*LRP2*	2
BTA3	109,426,963	8.62	*CSF3R*	-
BTA3	109,459,766	8.60	*OSCP1*	-
BTA3	109,773,240	8.42	*AGO3*	-
BTA3	14,998,951	7.89	*GON4L*	2
BTA3	34,201,135	7.64	*ELAPOR1*	-
BTA4	46,212,366	7.50	*KMT2E*	-
BTA4	97,342,492	7.32	*EXOC4*	15
BTA5	93,516,066	13.65	*MGST1*	11
BTA5	93,627,442	8.09	*SLC15A5*	5
BTA5	91,533,235	8.02	*PIK3C2G*	14
BTA5	94,720,983	7.93	*RERG*	6
BTA5	35,855,408	7.33	*NELL2*	9
BTA6	35,909,397	7.37	*FAM13A*	4
BTA8	108,975,271	9.09	*BRINP1*	-
BTA8	108,938,597	7.59	*BRINP1*	-
BTA10	44,811,185	7.77	*RTRAF*	-
BTA11	103,259,232	10.23	*PAEP*	70
BTA11	103,273,276	8.38	*GLT6D1*	1
BTA11	103,239,603	8.21	*ENSBTAG00000048091*	-
BTA11	75,068,176	8.16	*UBXN2A*	-
BTA12	68,572,875	7.84	*ENSBTAG00000054671*	-
BTA14	580,019	81.58	*SLC52A2*	1
BTA14	609,870	81.06	*DGAT1*	304
BTA14	550,784	80.98	*CPSF1*	12
BTA14	2,019,390	60.59	*TSNARE1*	35
BTA14	487,527	57.51	*CYHR1*	20
BTA15	74,629,039	7.99	*CD82*	-
BTA15	60,261,924	7.89	*KCNA4*	-
BTA16	66,282,178	8.69	*IVNS1APB*	-
BTA18	55,988,661	7.71	*RCN3*	-
BTA19	24,405,079	9.07	*ITGAE*	1
BTA19	23,765,194	7.39	*RAP1GAP2*	-
BTA20	32,018,037	14.27	*GHR*	168
BTA20	31,178,183	10.05	*NNT*	12
BTA20	31,373,420	9.36	*CCL28*	5
BTA20	33,341,878	7.54	*C6*	11
BTA20	29,362,772	7.53	*HCN1*	30
BTA21	9,641,396	7.48		-
BTA22	52,575	17.77	*ENSBTAG00000053760*	-
BTA23	13,850,106	8.09	*MOCS1*	16
BTA23	15,898,280	7.93	*GUCA1A*	2
BTA25	24,615,711	7.65	*C25H16orf82*	-
BTA26	42,681,843	7.35	*ENSBTAG00000051599*	-
BTA27	36,866,734	7.95	*KAT6A*	-
BTA27	36,466,414	7.78	*GINS4*	3
BTA27	25,562,534	7.37	*TNKS*	1
BTA28	36,164,984	7.34	*SH2D4B*	-
BTA29	48,340,327	8.46	*NADSYN*	-
BTA29	13,321,733	7.37	*ENSBTAG00000022427*	-

**Table 6:** Relevant biological functions for protein yield identified by functional annotation analyses in Holstein cows. Categories include: Biological process (BP), Cellular Component (CC), Molecular Function (MF).

GO	Term	*BH*/*FDR* *q*-Value	Genes	Biological Function
GO:1903494	Response to dehydroepiandrosterone	4.3 × 10^−2^	*CSN1S1*, *CSN2*	BP
GO:1903496	Response to 11-deoxycorticosterone	4.3 × 10^−2^	*CSN1S1*, *CSN2*	BP
GO:0051145	Differentiation of smooth muscle cells	4.6 × 10^−2^	*MRTFA*, *MRTFB*	BP
GO:0032570	Progesterone response	4.6 × 10^−2^	*CSN1S1*, *CSN2*	BP
GO:0032355	Response to estradiol	4.6 × 10^−2^	*CSN1S1*, *CSN2*	BP
GO:0005796	Golgi lumen	4.3 × 10^−2^	*CSN1S1*, *CSN2*	CC
GO:0016209	Antioxidant activity	4.6 × 10^−2^	*CSN1S1*, *CSN2*	MF
GO:0003713	Transcription coactivator activity	4.6 × 10^−2^	*MAML3*, *MRTFA*, *MRTFB*	MF

**Table 7:** Relevant biological functions for protein percentage identified by functional annotation analysis in Holstein cows. Categories include: Biological process (BP), Cellular Component (CC), Molecular Function (MF).

GO	Term	*BH*/*FDR* *q*-Value	Genes	Biological Function
GO:1903494	Response to dehydroepiandrosterone	0.00	*CSN1S1*, *CSN2*, *CSN3*	BP
GO:1903496	Response to 11-deoxycorticosterone	0.00	*CSN1S1*, *CSN2*, *CSN3*	BP
GO:0032570	Progesterone response	1.0 × 10^−3^	*CSN1S1*, *CSN2*, *CSN3*	BP
GO:0032355	Response to estradiol	1.0 × 10^−3^	*CSN1S1*, *CSN2*, *CSN3*	BP
GO:0045104	Organization of the filament cytoskeleton	3.4 × 10^−2^	*EPPK1*, *PLEC*	BP
GO:0005796	Golgi lumen	0.00	*CSN1S1*, *CSN2*, *CSN3*	CC
GO:0030056	Hemidesmosome	1.8 × 10^−2^	*EPPK1*, *PLEC*	CC
GO:0005794	Golgi apparatus	3.4 × 10^−2^	*CSN1S1*, *CSN2*, *CSN3*, *MAPK15*, *TRAPPC9*	CC
GO:0005576	Extracellular region	3.4 × 10^−2^	*CSN1S1*, *CSN2*, *CSN3*, *GHR*, *HSTN*	CC
GO:0005847	mRNA cleavage and polyadenylation specificity factor complex	3.4 × 10^−2^	*CPSF1*, *ZC3H3*	CC
GO:0005515	Protein binding	7.0 × 10^−3^	*CSN2*, *CSN3*, *SLC4A4*, *TRAPPC9*	MF
GO:0016209	Antioxidant activity	3.4 × 10^−2^	*CSN1S1*, *CSN2*	MF

**Table 8:** Relevant biological functions for fat percentage identified by functional annotation analysis in Holstein cows.

GO	Term	*BH*/*FDR* *q*-Value	Genes	Biological Function
GO:0051973	Upregulation of telomerase activity	4.8 × 10^−2^	*GRHL2*, *MAPK15*, *TNKS*	PB
GO:0031397	Negative regulation of protein ubiquitination	4.8 × 10^−2^	*VPS28*, *ADGRB1*, *IVNS1APB*	PB
GO:0035279	mRNA cleavage involved in gene silencing by miRNA	4.8 × 10^−2^	*AGO2*, *AGO3*	PB
GO:0030425	Dendrite	2.1× 10^−2^	*BRINP1*, *LRP2*, *ADGRB1*, *AGO2*, *HCN1*, *PLEC*	CC
GO:0005829	Cytosol	4.8 × 10^−2^	*RAP1GAP2*, *RERG*, *UBXN2A*, *VPS28*, *AGO2*, *AGO3*, *EEF1D*, *GPAA1*, *GHR*, *IVNS1APB*, *KAT6A*, *MOCS1*, *PLEC*, *PTK2*, *STK40*, *SPAG1*, *TNKS*	CC
GO:0090624	Endoribonuclease activity, cleaving mRNA paired with miRNA	4.8 × 10^−2^	*AGO2*, *AGO3*	MF

## Data Availability

The datasets presented in this article are not readily available due to data ownership restrictions, as they are part of a proprietary dataset from an industry collaborator.

## Appendix A

**Table A1:** Relevant biological functions for protein yield identified by functional annotation analyses in Holstein cows (non-significant *q*-values). Categories include: Biological process (BP), Cellular Component (CC), Molecular Function (MF).

GO	Term	*BH*/*FDR* *q*-Value	Genes	Biological Function
GO:0010467	Gene expression	8.9 × 10^−2^	*PLEC*, *SVEP1*	BP
GO:0098869	Oxidizing cellular detoxification	8.9 × 10^−2^	*CSN1S1*, *CSN2*	BP

**Table A2:** Relevant biological functions for protein percentage identified by functional annotation analysis in Holstein cows (non-significant *q*-values). Categories include: Biological process (BP), Cellular Component (CC), Molecular Function (MF).

GO	Term	*BH*/*FDR* *q*-Value	Genes	Biological Function
GO:0031397	Negative regulation of protein ubiquitination	7.5 × 10^−2^	*VPS28*, *ADGRB1*	BP
GO:0042060	Wound healing	7.8 × 10^−2^	*EPPK1*, *PLEC*	BP
GO:0098869	Oxidizing cellular detoxification	7.8 × 10^−2^	*CSN1S1*, *CSN2*	BP

**Table A3:** Relevant biological functions for fat yield identified by functional annotation analysis in Holstein cows (non-significant *q*-values). Categories include: Biological process (BP), Cellular Component (CC), Molecular Function (MF).

GO	Term	*BH*/*FDR* *q*-Value	Genes	Biological Function
GO:0030163	Catabolic process of cellular proteins	8.1 × 10^−2^	*DENND3*, *PPT1*	BP
GO:0045104	Organization of the intermediate filament cytoskeleton	8.1 × 10^−2^	*EPPK1*, *PLEC*	BP
GO:0048741	Skeletal muscle fiber development	8.1 × 10^−2^	*ACTA2*, *PLEC*	BP
GO:0008344	Adult locomotor behavior	8.7 × 10^−2^	*EPS8*, *PPT1*	BP
GO:0031397	Negative regulation of protein ubiquitination	8.7 × 10^−2^	*VPS28*, *ADGRB1*	BP
GO:0030425	Dendrite	8.1 × 10^−2^	*ADGRB1*, *AGO2*, *PPT1*, *PLEC*	CC
GO:0030056	Hemidesmosome	8.1 × 10^−2^	*EPPK1*, *PLEC*	CC
GO:0005856	Cytoskeleton	8.1 × 10^−2^	*ARHGAP39*, *PLEC*, *PTK2*, *PTPN14*	CC
GO:0005737	Cytoplasm	8.1 × 10^−2^	*IQANK1*, *MAF1*, *RBM19*, *ARHGAP39*, *VPS28*, *ACTA2*, *AHSP*, *EPS8*, *EPPK1*, *LDHB*, *MAPK15*, *PIK3C2G*, *PLEC*, *PTPN14*	CC
GO:0005925	Focal adhesion	8.1 × 10^−2^	*ADGRB1*, *PLEC*, *PTK2*	CC
GO:000584	mRNA cleavage and polyadenylation specificity factor complex	8.1 × 10^−2^	*CPSF1*, *ZC3H3*	CC
GO:0005829	Cytosol	8.1 × 10^−2^	*KIAA0753*, *RERG*, *VPS28*, *AGO2*, *CMYA5*, *EEF1D*, *GPAA1*, *PPT1*, *PLEC*, *PTK2*, *SPIN1*	CC
GO:0048471	Perinuclear region of the cytoplasm	8.1 × 10^−2^	*ADGRB1*, *CYHR1*, *EXOC3*, *PLEC*	CC
GO:0005794	Golgi apparatus	8.1 × 10^−2^	*RAB2A*, *EXOC3*, *MAPK15*, *PPT1*, *TRAPPC9*	CC
GO:0030424	Axon	8.1 × 10^−2^	*PPT1*, *PLEC*, *SEMA3A*	CC
GO:0005903	Brush edge	8.5 × 10^−2^	*EPS8*, *PLEC*	CC
GO:0003779	Actin binding	8.5 × 10^−2^		MF

**Table A4:** Relevant biological functions for fat percentage identified by functional annotation analysis in Holstein cows (non-significant *q*-values). Categories include: Biological process (BP), Cellular Component (CC), Molecular Function (MF).

GO	Term	*BH*/*FDR* *q*-Value	Genes	Biological Function
GO:0060396	Growth hormone receptor signaling pathway	6.2 × 10^−2^	*GHR*, *PTK2*	PB
GO:0070922	miRNA loading in RISC involved in gene silencing by miRNA	6.4 × 10^−2^	*AGO2*, *AGO3*	PB
GO:0010501	Unwinding of RNA secondary structure	6.5 × 10^−2^	*AGO2*, *AGO3*	PB
GO:0035264	Growth of multicellular organism	6.7 × 10^−2^	*GRHL2*, *PLEC*, *STK40*	PB
GO:0035278	miRNA-mediated translation inhibition	6.7 × 10^−2^	*AGO2*, *AGO3*	PB
GO:0031054	Pre-miRNA processing	6.9 × 10^−2^	*AGO2*, *AGO3*	PB
GO:1904355	Upregulation of telomere capping	7.3 × 10^−2^	*MAPK15*, *TNKS*	PB
GO:0045104	Organization of the intermediate filament cytoskeleton	7.4 × 10 ^−2^	*EPPK1*, *PLEC*	PB
GO:0030056	Hemidesmosome	6.2 × 10^−2^	*EPPK1*, *PLEC*	CC
GO:0070578	RISC Charging Complex	6.4 × 10^−2^	*AGO2*, *AGO3*	CC
GO:0016020	Membrane	6.7 × 10^−2^	*EPPK1*, *GLT6D1*, *GHR*, *GUCA1A*, *PIK3C2G*, *PLEC*, *TSNARE1*	CC
GO:0016442	RISC Complex	6.7 × 10^−2^	*AGO2*, *AGO3*	CC
GO:0005783	Endoplasmic reticulum	7.3 × 10^−2^	*BRINP1*, *LRP2*, *UBXN2A*, *MGST1*, *RCN3*, *TRAPPC9*	CC
GO:0005847	mRNA cleavage and polyadenylation specificity factor complex	7.3 × 10^−2^	*CPSF1*, *ZC3H3*	CC
GO:0005925	Focal adhesion	8.2 × 10^−2^	*ADGRB1*, *PLEC*, *PTK2*	CC
GO:0009897	Outer side of the plasma membrane	8.4 × 10^−2^	*LRP2*, *CSF3R*, *GHR*, *ITGAE*	CC
GO:0005515	Protein binding	6.4 × 10^−2^	*GUCA1A*, *NCALD*, *PAEP*, *TRAPPC9*	MF
GO:0046872	Metal ion binding	7.3 × 10 ^−2^	*FBXO43*, *AGO2*, *AGO3*, *CYHR1*, *ITGAE*, *KMT2E*, *MOCS1*, *OSR2*, *RNF19A*, *ZC3H3*	MF
GO:0035198	miRNA binding	8.2 × 10^−2^	*AGO2*, *AGO3*	MF
GO:0000993	Binding to the core of RNA polymerase II	8.4 × 10^−2^	*AGO2*, *AGO3*	MF
